# Cost-effectiveness analysis of offering free leisure centre memberships to physically inactive members of the public receiving state benefits: a case study

**DOI:** 10.1186/s12889-016-3300-x

**Published:** 2016-07-22

**Authors:** Talitha I. Verhoef, Verena Trend, Barry Kelly, Nigel Robinson, Paul Fox, Stephen Morris

**Affiliations:** Department of Applied Health Research, University College London, Gower Street, London, WC1E 6BT UK; Camden Borough Council, London, UK; London Sport, London, UK

**Keywords:** Physical activity, Mental wellbeing, Cost-utility analysis, Health economics

## Abstract

**Background:**

We evaluated the cost-effectiveness of the Give-it-a-Go programme, which offers free leisure centre memberships to physically inactive members of the public in a single London Borough receiving state benefits.

**Methods:**

A decision analytic Markov model was developed to analyse lifetime costs and quality-adjusted life-years (QALYs) of 1025 people recruited to the intervention versus no intervention. In the intervention group, people were offered 4 months of free membership at a leisure centre. Physical activity levels were assessed at 0 and 4 months using the International Physical Activity Questionnaire (IPAQ). Higher levels of physical activity were assumed to decrease the risk of coronary heart disease, stroke and diabetes mellitus type II, as well as improve mental health. Costs were assessed from a National Health Service (NHS) perspective. Uncertainty was assessed using one-way and probabilistic sensitivity analyses.

**Results:**

One-hundred fifty nine participants (15.5 %) completed the programme by attending the leisure centre for 4 months. Compared with no intervention, Give it a Go increased costs by £67.25 and QALYs by 0.0033 (equivalent to 1.21 days in full health) per recruited person. The incremental costs per QALY gained were £20,347. The results were highly sensitive to the magnitude of mental health gain due to physical activity and the duration of the effect of the programme (1 year in the base case analysis). When the mental health gain was omitted from the analysis, the incremental cost per QALY gained increased to almost £1.5 million. In the probabilistic sensitivity analysis, the incremental costs per QALY gained were below £20,000 in 39 % of the 5000 simulations.

**Conclusions:**

Give it a Go did not significantly increase life-expectancy, but had a positive influence on quality of life due to the mental health gain of physical activity. If the increase in physical activity caused by Give it a Go lasts for more than 1 year, the programme would be cost-effective given a willingness to pay for a QALY of £20,000.

**Electronic supplementary material:**

The online version of this article (doi:10.1186/s12889-016-3300-x) contains supplementary material, which is available to authorized users.

## Background

Physical inactivity is associated with morbidity and mortality. Several studies have demonstrated the protective effect of regular physical activity on risk of many chronic diseases, such as diabetes mellitus, coronary heart disease and stroke [1–3]. General health and quality of life are also increased in people who undertake regular physical activity [4]. However, only 3 in 10 individuals in England undertake a minimum of 30 min of moderate intensity physical activity on at least 5 days per week, as recommended by the UK Department of Health [5].

The London Borough of Camden, through the Pro-Active Camden Partnership, started the Give it a Go project to tackle inactivity within the Borough by reducing cost as a barrier to participation. The project was targeted at inactive individuals (defined as those with a sedentary job and no physical exercise or cycling) or moderately inactive individuals (defined as those with a sedentary job and <1 h physical exercise and /or cycling or with a standing job and no physical exercise or cycling) attending the National Health Service (NHS) Health Checks programme and people in receipt of state benefits. The London Borough of Camden is the 15^th^ most deprived Borough within London and has the third greatest health inequalities across London. Give it a Go offers free leisure centre membership to physically inactive people and was first launched in 2009 and the first scheme recruited 1775 Camden residents retaining 82 % after month 1 with 22 % of partipants completing the scheme (which means they attended the leisure centre for 4 months). Subsequent Give it a Go schemes based on the same model but informed by learning from each of the previous schemes have seen over 4000 people join Give it a Go with average completion rates for the scheme at 22 %. Give it a Go is now a mainstream physical activity intervention in Camden. It is important to assess the cost-effectiveness of the project as well as its effectiveness, to make decisions about the future use of available money for projects such as these. Evaluation of the cost-effectiveness of new projects is important to inform service delivery, particularly in state funded healthcare systems such as the United Kingdom’s (UK) National Health Service (NHS). The aim of the present study was therefore to study the cost-effectiveness (in terms of the incremental cost per quality-adjusted life-year (QALY) gained) of the third (most recent) incarnation of the Give it a Go scheme.

## Methods

### Model structure

**Fig. 1:**
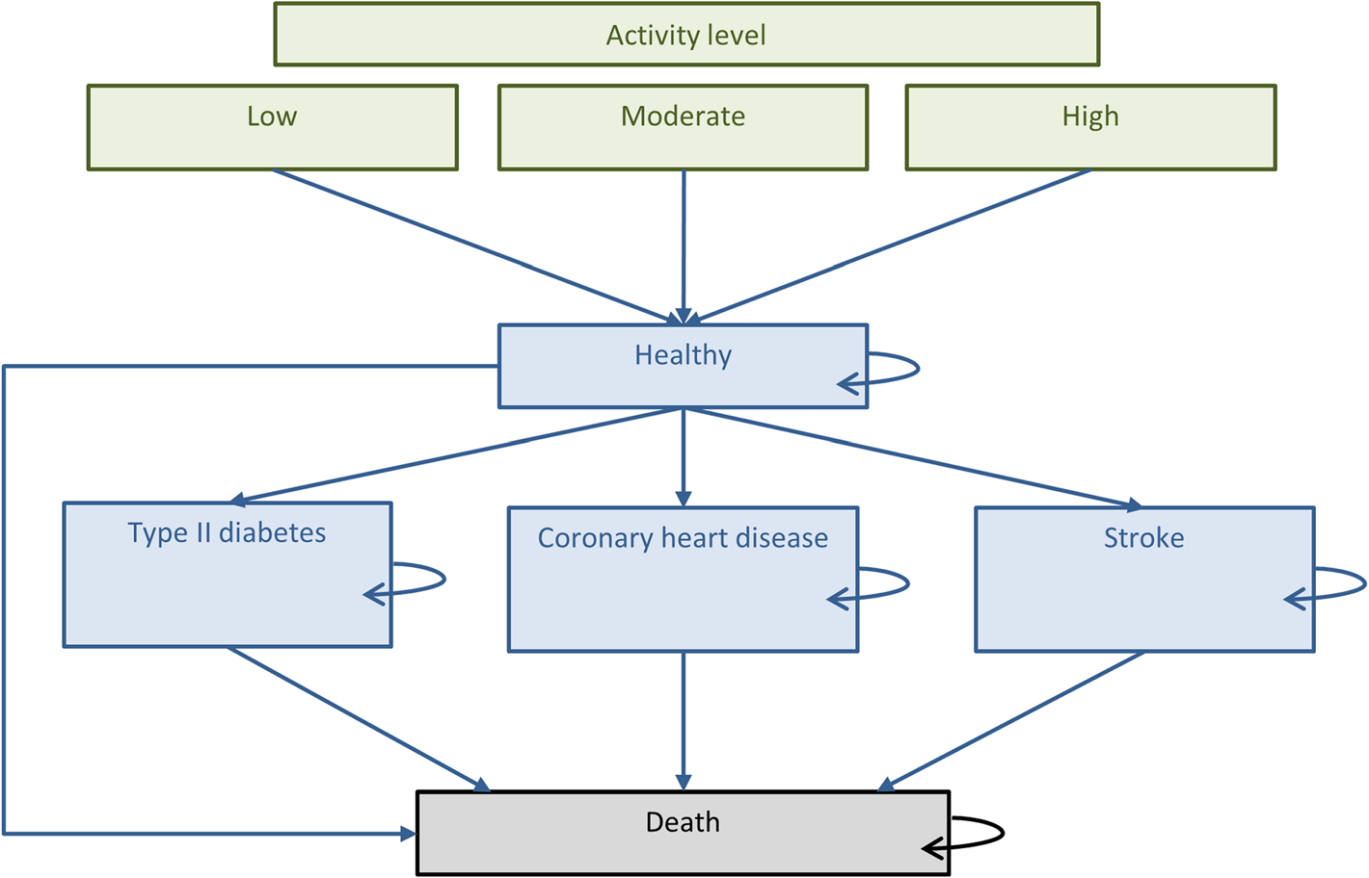
Markov model

To analyse the costs and QALYs of the Give it a Go project compared to a situation without Give it a Go, we developed a decision analytic Markov model. Figure 1 depicts the possible pathways in the Markov model. The model was based on a previous model developed to assess the cost-effectiveness of an intervention to improve physical activity [6]. In the model all people start in the ‘healthy’ state. Depending on their physical activity level, they have different chances of developing one of three diseases (type II diabetes, stroke or coronary heart disease). When a disease occurs, people are expected to stay in that state until they die. Once a month, people can move from healthy to a disease state or dead or stay in the same health state. The model has a lifetime time horizon, running until all patients are in the absorbing ‘death’ state. The starting age of the cohort was 45 years, based on the average age of people recruited for the Give it a Go programme. We varied the starting age from 35 to 55 years in sensitivity analyses. Average age, activity levels, compliance to the scheme and costs of the scheme were taken from the Give it a Go evaluation, all other input parameters were taken from the literature.

### Intervention

People who could participate in Give it a Go 2013 were residents of the London Borough of Camden who were 18 years of age or above and who were in receipt of a minimum of one form of state benefit or had attended an NHS Health Check and been identified as being inactive or moderately inactive through the General Practice Physical Activity Questionnaire (GPPAQ). They were not excluded if they had diabetes, stroke or coronary heart disease, but in the model we assumed all participants to be healthy at the start. Participants were recruited through a referral letter from the Health Check programme or through a mailout to all residents of Camden who recieved state benefits. People were excluded if they were already members of a leisure facility or if they did not have written authorisation from their GP to participate in the programme due to medical conditions identified on the Physical Activity Readiness Questionnaire. The programme was provided at five leisure centres in Camden. Participants could go to the leisure centre of their choice for an initial one hour induction meeting and received a free 4 month membership. Each month, the participant had to attend a minimum of five times in order to qualify for the next month. Several incentives were created to increase uptake of the programme, such as prize draws or the opportunity to bring a friend.

### Physical activity level

In total, 1025 people were recruited (and registered for an induction meeting) to the programme. All participants completed the short version of the International Physical Activity Questionnaire (IPAQ) at baseline (prior to first attendance). The IPAQ questionnaire included questions on how much time was spent on vigorous and moderate physical activities and walking during the past 7 days. The results of this questionnaire were used to categorize baseline activity level into low, moderate and high (see Additional file 1). The proportion of people in each level at baseline measurement was used for the comparator group and for the participants not completing the programme. The programme was completed (attended the leisure centre ≥5 times per month for 4 months) by 159 participants (15.5 %). Only 52 of these completed the follow-up IPAQ questionnaire, which was to be completed at the end of the 4 month period. The proportion of these 52 participants in each activity level was assumed to be the same for all 159 completers. Baseline activity level of the completers only (with and without follow-up data) is shown in the Additional file 1: Table S1. We assumed that the physical activity level of all but the completers remained at the baseline level during the 4 month period.

Since we used a lifetime time horizon, it is important to consider the expected duration of the increased activity levels among completers. If the increased activity would only last for the 4 month duration of the programme the health benefits will probably be small; if increased activity is maintained for the whole lifetime the health benefits will probably be large. No data regarding the long term effects on physical activity beyond the duration of the programme were collected. We assumed that completers of the programme would stay in their activity level at the end of the programme (low, moderate, high) for 12 months in total (so another 8 months after completing the programme), but we varied this value from 4 to 20 months in the sensitivity analysis, to account for uncertainty around this parameter. Activity levels (baseline and follow-up) and other input parameters for the model are shown in Tables 1 and 2.

**Table 1:** General input parameters

	Base case value	Lower limit	Upper limit	Distribution for PSA	Source
Starting age	45	25	65	Normal	GIAG
Proportion of recruited participants completing the programme	15.5 %	1 %	50 %	Beta	GIAG
Baseline activity level-all recruited participants					
Low	32.4 %	–	–	Dirichlet	GIAG
Moderate	37.6 %	25 %	50 %	Dirichlet	GIAG
High	30.0 %	15 %	50 %	Dirichlet	GIAG
Follow-up activity level-completers					
Low	23.5 %	–	–	Dirichlet	GIAG
Moderate	23.5 %	10 %	40 %	Dirichlet	GIAG
High	52.9 %	35 %	65 %	Dirichlet	GIAG
Duration of effect of Give it a Go on PA (months)	12	4	20	Uniform	Assumption
Relative risks of coronary heart disease					
Moderate activity level	0.90	0.83	0.99	Lognormal	[1]
High activity level	0.81	0.68	0.96	Lognormal	[1]
Relative risks of stroke					
Moderate activity level	0.86	0.79	0.93	Lognormal	[3]
High activity level	0.74	0.64	0.85	Lognormal	[3]
Relative risks of type II diabetes					
Moderate activity level	0.67	0.53	0.84	Lognormal	[2]
High activity level	0.61	0.41	0.9	Lognormal	[2]
Relative risk for mortality					
Non CVD mortality after CHD	1.71	1.44	1.98	Lognormal	[14]
CVD mortality after CHD	3.89	3.81	3.97	Lognormal	[14]
Non CVD mortality after stroke	1.71	1.44	1.98	Lognormal	[14]
CVD mortality after stroke	3.89	3.81	3.97	Lognormal	[14]
Non CVD mortality after diabetes	1.49	1.24	1.74	Lognormal	[15]
CVD mortality after diabetes	2.61	2.34	2.88	Lognormal	[15]
Utilities					
CHD 1st event	0.80	0.622	0.931	Beta	[6]
Post CHD first event	0.92	0.665	1.000	Beta	[6]
Stroke 1st event	0.63	0.503	0.749	Beta	[6]
Post stroke 1st event	0.65	0.518	0.771	Beta	[6]
Diabetes	0.90	0.665	0.997	Beta	[6]
Mental health gain when moderately active	0.023	0.000	0.200	Beta	[8]
Mental health gain when highly active	0.104	0.000	0.200	Beta	[8]

*Abbreviations*: *GIAG* Give it a Go, *PA* physical activity, *CVD* cardiovascular disease, *CHD* coronary heart disease

**Table 2:** Age specific input parameters [6]

	Base case value	Lower limit	Upper limit	Distribution for PSA
Incidence rates (yearly)				
CHD 33–34	0.0035 %	0.0017 %	0.0059 %	Beta
CHD 35–44	0.0465 %	0.0391 %	0.0546 %	Beta
CHD 45–54	0.2095 %	0.1933 %	0.2263 %	Beta
CHD 55–65	0.6310 %	0.6028 %	0.6599 %	Beta
CHD 65–74	0.9700 %	0.9350 %	1.0056 %	Beta
CHD 75–81	0.9700 %	0.9350 %	1.0056 %	Beta
Stroke 33–34	0.0080 %	0.0035 %	0.0142 %	Beta
Stroke 35–44	0.0230 %	0.0148 %	0.0330 %	Beta
Stroke 45–54	0.0570 %	0.0435 %	0.0723 %	Beta
Stroke 55–65	0.2910 %	0.2593 %	0.3245 %	Beta
Stroke 65–74	0.6900 %	0.6408 %	0.7410 %	Beta
Stroke 75–81	1.4340 %	1.3630 %	1.5068 %	Beta
Diabetes 33–39	0.0090 %	0.0077 %	0.0104 %	Beta
Diabetes 40–49	0.0280 %	0.0257 %	0.0305 %	Beta
Diabetes 50–59	0.0632 %	0.0596 %	0.0669 %	Beta
Diabetes 60–69	0.1005 %	0.0959 %	0.1051 %	Beta
Diabetes 70–79	0.1116 %	0.1068 %	0.1164 %	Beta
Diabetes 80–81	0.1116 %	0.1068 %	0.1164 %	Beta
Probability of event being fatal				
CHD fatal 33–34	8.77 %	7.130 %	10.566 %	Beta
CHD fatal 35–44	8.77 %	7.130 %	10.566 %	Beta
CHD fatal 45–54	8.77 %	7.130 %	10.566 %	Beta
CHD fatal 55–65	11.55 %	9.386 %	13.910 %	Beta
CHD fatal 65–74	21.07 %	17.087 %	25.336 %	Beta
CHD fatal 75–81	14.76 %	11.988 %	17.769 %	Beta
Stroke fatal 33–34	23.46 %	19.024 %	28.212 %	Beta
Stroke fatal 35–44	23.46 %	19.024 %	28.212 %	Beta
Stroke fatal 45–54	23.46 %	19.024 %	28.212 %	Beta
Stroke fatal 55–65	23.28 %	18.876 %	27.991 %	Beta
Stroke fatal 65–74	23.47 %	19.026 %	28.215 %	Beta
Stroke fatal 75–81	23.42 %	18.989 %	28.160 %	Beta
Utility weights in low activity but healthy population				
Age 33–44	0.90	0.880	0.919	Beta
Age 45–54	0.86	0.840	0.879	Beta
Age 55–64	0.82	0.800	0.839	Beta
Age 65–74	0.78	0.760	0.799	Beta
Age 75+	0.72	0.700	0.739	Beta

*Abbreviations*: *CHD* coronary heart disease

### Disease risk

It has been shown that increased physical activity can improve health by reducing the risk of several diseases. In this model we included the following diseases: Type II diabetes, coronary heart disease and stroke. We used data from the study by Anokye et al. [6] to assign an age-specific disease risk to all people with a low activity level. More active people will have a lower disease risk which is reflected in a relative risk of moderate vs low activity level and high vs low activity level. The risk reduction was only assumed for the time the person was more active (no residual protective effect). We assumed that a proportion of strokes and coronary heart disease events would be immediately fatal. When people survived a stroke or coronary heart disease event, their subsequent risk of cardiovascular disease (CVD) related mortality, as well as non-CVD related mortality was increased. Age specific probabilities for CVD related mortality and non-CVD related mortality were derived from UK life tables and causes of death prepared by the Office of National Statistics.

### Quality of life

To estimate the quality-adjusted life-years (QALYs) in both the intervention and the comparator groups we used data on health related quality of life for each of the health states to weight the survival years. Age specific quality of life was assessed for all participants, because quality of life is assumed to fall with age. A disease-independent effect of physical activity on quality of life can be expected. In an analysis performed by NICE on promoting physical activity [7], it was estimated that every 30 min of physical activity results in a QALY gain of 0.000222433333 due to improvements in mental wellbeing brought about by partaking in physical activity, based on an earlier study on the cost-effectiveness of environmental interventions to promote physical activity [8]. Using the data collected in this study, we found that people with moderate activity levels spend on average 1 h per week more on physical activity than people with low activity levels and people with high activity levels spend on average 4.5 h per week more on physical activity. Applying the QALY gain of 0.000222433333 per 30 min to every extra half an hour spent on physical activity over 1 year, we estimated the QALY gain per year of physical activity in the moderate activity group and in the high activity group. These utility values for mental health gain were varied over a wide range in the sensitivity analysis.

### Costs

The perspective of this study was that of the NHS and Personal Social Services. The costs of the Give it a Go programme were divided into general costs (incurred for the entire recruited group) and sports related costs (full costs are incurred for every completer, but non-completers also incur some costs). The general costs of the programme consisted of costs for evaluation, communication, mail out for recruitment, training, project coordination and incentives. Total general costs were £39,255 or £38.30 per recruited person. Sports related costs consisted of induction costs (£20), membership per month (only for month 1&4, the leisure centres provided month 2&3, £28 per month), follow-up appointment (£20) and communication costs (£1). Total sports related costs were therefore £97 per completer. Because some non-completers incurred no costs at all (they did not start the programme, *n* = 300) and some incurred almost all costs (a further 280 participants dropped out before completing month 1, 138 before completing month 2, 107 before completing month 3 and 41 before completing month 4), we calculated that the average costs per non-completer were £16.75. The leisure centre membership was paid for 2 months by the programme and actual use of services did not influence these costs.

We also considered costs relevant to the NHS related to the 3 diseases. We used the same costs for coronary heart disease, stroke and diabetes as in a previous study on the cost effectiveness of improving physical activity [6], but inflated the costs to 2013/14 UK£ (Table 3). All costs (presented in 2013/14 UK£) and health outcomes were discounted using a discount rate of 3.5 % per year [9].

**Table 3:** Costs

	Base case value	Lower limit	Upper limit	Distribution for PSA	Source
GIAG general - per person recruited	£38	£10	£100	Gamma	GIAG
GIAG leisure - per completer	£97	£50	£150	Gamma	GIAG
GIAG leisure - per non-completer^a^	£17	£5	£50	Gamma	GIAG
CHD 1st event	£4,144	£3,372	£4,995	Gamma	[6]
Post CHD first event, per year	£473	£385	£571	Gamma	[6]
Stroke 1st event	£10,698	£8,704	£12,894	Gamma	[6]
Post stroke 1st event, per year	£2,350	£1,912	£2,832	Gamma	[6]
Diabetes, per year	£955	£777	£1,152	Gamma	[6]

*Abbreviations*: *GIAG* Give it a Go, *CHD* coronary heart disease

^a^This is an average for all non-completers. Some some non-completers did not participate at all, while others participated a week to a few months

### Model outputs and sensitivity analysis

The main outcome of the model was the incremental costs per QALY gained for Give it a Go versus no intervention, using the base case values for every parameter. There were many uncertainties and assumptions in this model. These include the duration of the increased physical activity, the association between physical activity and disease risk and the association between physical activity and mental health. We therefore performed several sensitivity analyses. First, we varied each parameter one at a time over a plausible range and examined if the main outcome varied appreciably (changed from below £20,000-a willingness to pay threshold of £20,000 per QALY gained as recommended by the National Institute of Health and Care Excellence (NICE) [9]-to above this value or vice versa) in a one-way sensitivity analysis. Second, a probabilistic sensitivity analysis was performed, producing 5000 simulations of the outputs based on drawing random samples from the probability distributions of all input parameters, to calculate the chance that Give it a Go would be cost-effective at different levels of the willingness-to-pay per QALY gained and draw a cost-effectiveness acceptability curve. Beta distributions were used for probabilities and utilities, log-normal distributions for relative risks and gamma distributions for all costs.

## Results

### Base case

We estimated that the QALY gain per year of physical activity in the moderate activity group was 0.02 and in the high activity group 0.10. Table 4 shows the total costs of Give it a Go versus no intervention as well as the total life-years and QALYs. Mean costs per person were increased by £67.25, which is effectively the costs of the Give it a Go programme (the programme increased costs by £67.49 and NHS costs for treating diseases were decreased by £0.25). Life-years were increased by only 0.0001 (0.02 days) and QALYs were increased by 0.0033 (1.21 days in full health). The incremental costs per QALY gained were therefore £20,347. The increase in physical activity caused by the Give it a Go programme, did not result in significant differences in incidence of coronary heart disease, stroke or diabetes incidence (see Additional file 1).

**Table 4:** Base case results on costs, life-years and QALYs of Give it a Go versus no intervention

	Total costs (95 % CI)	Total life-years (95 % CI)	Total QALYs (95 % CI)	Incremental costs per QALY gained (95 % CI)
No intervention	£2,287 (1,428–3,312)	19.235 (16.233–21.758)	16.370 (13.255–19.325)	–
Give it a Go	£2,354 (1,495–3,374)	19.235 (16.234–21.758)	16.373 (13.257–19.328)	–
Increment	£67.25 (48–90)	0.0001 (0.0000–0.0002)	0.0033 (0.0002–0.0106)	£20,347 (513–35,119)

*Abbreviations*: *QALY* quality-adjusted life-year, *CI* Confidence interval

### One-way sensitivity analysis

In the one-way sensitivity analysis most parameters, when varied over the specified range, did not change the results from an incremental cost above £20,000 per QALY gained to below £20,000 per QALY gained. The incremental costs per QALY gained would be below this threshold if the average age of participant would be 55 years or more, if 15.8 % or more of the recruited people would complete the programme, if only 36.1 % or less or 29.7 % or less of the people in the control group would already have a moderate or high activity level respectively, if 25.0 % or more or 53.3 % or more of the people completing the programme would have a moderate or high activity level respectively, or if the increased activity level would exist 13 months or more after the intervention (see Table 5). Also, a very large influence of the mental health gained by physical activity was seen. The incremental costs per QALY gained would be below the threshold if the mental health gain when moderately active would be 0.021 or less QALYs per year or if the mental health gain when highly active would be 0.106 or more. If no mental health gain would be included for increased physical activity, the incremental costs per QALY gained would be almost £1.5 million. The incremental costs per QALY gained would also be below £20,000 if the costs of the Give it a Go programme would be £1 to £7 lower.

**Table 5:** One-way sensitivity analysis. Parameters that had a large influence on the cost-effectiveness ratio in the one-way sensitivity analysis (changing the results from an incremental cost above £20,000 per QALY gained to below £20,000 per QALY gained) when varied over the specified range

	Base case value	Threshold value^a^
Starting age	45	55
Proportion of recruited participants completing the programme	15.5 %	15.8 %
Baseline activity level-all recruited participants		
Moderate	37.6 %	36.1 %
High	30.0 %	29.7 %
Follow-up activity level-completers		
Moderate	23.5 %	25.0 %
High	52.9 %	53.3 %
Duration of effect of Give it a Go on PA (months)	12	13
Utilities		
Mental health gain when moderately active	0.023	0.021
Mental health gain when highly active	0.104	0.106
Costs		
GIAG general-per person recruited	£38.30	£37.15
GIAG leisure-per completer	£97.00	£89.60
GIAG leisure-per non-completer	£16.75	£15.39

^a^The threshold value is the value at which the ICER would be £20,000

### Probabilistic sensitivity analysis

**Fig. 2:**
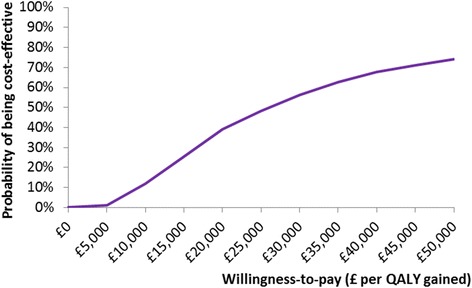
Cost-effectiveness acceptability curve. This graph shows the probability that Give it a go would be cost-effective at different willingness to pay thresholds

In the probabilistic sensitivity analysis, the incremental costs per QALY gained were below £20,000 in 39 % of the simulations and below £30,000 in 56 % of the simulations. The probability that give it a go would be cost-effective at different thresholds of willingness to pay is shown in Fig. 2.

## Discussion

### Main findings

Give it a Go did not significantly increase life-expectancy, but had a positive influence on quality of life due to the mental health gain of physical activity. If the increase in physical activity caused by Give it a Go lasts for more than 1 year, the programme would be cost-effective given a willingness to pay for a QALY of £20,000.

### Comparisons with other studies

In previous studies on the cost-effectiveness of schemes to improve physical activity, similar results were found. Anokye et al. found that brief advice was a cost-effective way to promote physical activity in primary care [6]. If mental health gains of physical activity were included, the costs per QALY gained were £1730, but if these mental health gains were excluded the costs per QALY gained exceeded the £20,000 threshold. In another study, the cost-effectiveness of exercise referral schemes was assessed [10]. These schemes had an incremental cost-effectiveness ratio of £20,876. Frew et al. studied the cost-effectiveness of a programme similar to Give it a Go (free access to leisure centres at certain times of the day) and found that the incremental costs of this programme were £400 per QALY gained [11]. In this study, the authors used a time horizon of 5 years in the base case analysis, assuming the increased physical activity was maintained during this entire period.

### Strengths and limitations

The compliance to the programme was less than 16 % and only one third of the people completing the programme filled in the follow-up questionnaire. This low response rate presents an important data limitation. Another limitation of this study is that there were no long term data on physical activity maintenance beyond the end of the programme. We assumed that for people not completing the programme, their activity level would stay at the same level as at the start of the programme and for people completing the programme, their activity level would stay at the same level as at the moment of completing the programme (for 12 months in the base case analysis). However, of the 159 people completing the programme, only 42 took up a membership at the leisure centre. It is possible that the activity level of the remaining 117 people would fall back to the baseline level, though they may have continued to exercise by other means. On the other hand, we might have underestimated the benefits of the programme by assuming that the physical activity level of people who only participated for 1 to 3 months did not change, while they still might have accrued some benefit during that period or even stayed more active, but outside the Give it a Go programme. There might also be an impact on people invited for the programme who decided not to participate. These people might have been prompted to increase their physical activity level, but decided to do this outside the programme. If this would have been the case, the cost-effectiveness of the programme could have been underestimated.

Another limitation is the uncertainty around the mental health gain of physical activity. If there is no mental health gain related to physical activity, the health gain of Give it a Go would be very small and incremental costs per QALY gained are very high. We used an estimate of 0.000222433333 per 30 min of increased physical activity compared to people with a low activity level. For people with a moderate activity level this was 0.023 and for people with a high activity level this was 0.104. This is comparable to a previous study in which the authors used a value of 0.07 in all active persons compared with inactive persons [6]. Using the estimate of 0.0002222433333 per 30 min of increased physical activity in the present study, we assumed the additional QALY gains to be linear over time. However, in reality additional minutes of physical activity may result in an increase in QALYs, but at a decreasing rate. This might have led to overestimating the QALY gain in people with a high activity level. In future studies, the health gain of increased physical activity could be investigated by using a questionnaire on quality of life, such as the EuroQol-5 Dimensions (EQ-5D) [12]. This way, the increase in quality of life caused by physical activity could be assessed more accurately. Even though this questionnaire might not be very sensitive to changes in mental health, this would be a more accurate method than the indirect method used in this study.

In this model we included only three diseases (type II diabetes, coronary heart disease and stroke). There are other diseases that are associated with a lack of physical activity as well (e.g. breast cancer, musculoskeletal conditions, depression), but we did not include these in our model. This narrow disease range might lead us to under- or over-estimate the cost-effectiveness of the programme. In order not to overcomplicate the model, we assumed all subjects were healthy at the start, which also might lead us to under- or over-estimate the cost-effectiveness of the programme. The incidence of the three diseases included in this study were taken from the study of Anokye et al. [6]. In a different study, the incidence of type 2 diabetes in the UK was shown to be somewhat higher (515 per 100,000) [13]. This might lead us to underestimate the effect of the Give it a Go programme.

The Give it a Go scheme included 4 months of free leisure centre membership, of which 2 months were paid for by the leisure centre. This means that if these costs cannot be borne by the leisure centre, this would add to the costs of the programme, which would in turn be less cost-effective.

## Conclusion

In conclusion, provision of free leisure centre membership to physically inactive members of the public receiving state benefits or those identified as being inactive via NHS Health Checks could represent good value for money, but is highly dependent on the long-term effects of leisure centre membership on physical activity, uptake and compliance to the scheme and the impact of physical activity on mental wellbeing.

## Abbreviations

CHD, Coronary Heart Disease; CI, Confidence Interval; CVD, Cardiovascular Disease; EQ-5D, EuroQol 5 Dimensions; GIAG, Give it a Go; GP, General Practitioner; GPPAQ, General Practice Physical Activity Questionnaire; IPAQ, International Physical Activity Questionnaire; NHS, National Health Service; NICE, National Institute of Health and Care Excellence; PA, Physical Activity; QALY, Quality-adjusted Life-Year

## Additional file

Additional file 1: Supplement to: Cost-effectiveness of offering free leisure centre memberships to physically inactive members of the public receiving state benefits. (PDF 561 kb)
