# Supplementary material for: Cost-effectiveness analysis of offering free leisure centre memberships to physically inactive members of the public receiving state benefits: a case study
Source: BMC Public Health. 2016 Jul 22;16:616. doi: 10.1186/s12889-016-3300-x (PMC4957286; doi:10.1186/s12889-016-3300-x)
Supplement: Additional file 1: — Supplement to: Cost-effectiveness of offering free leisure centre memberships to physically inactive members of the public receiving state benefits. (PDF 561 kb) [file 12889_2016_3300_MOESM1_ESM.pdf]

Supplement to:

Cost-effectiveness of offering free leisure centre memberships to physically inactive members of the public receiving state benefits

Talitha I. Verhoef[1], Verena Trend[2], Barry Kelly[2,3], Nigel Robinson[2], Paul Fox[2] and Steve Morris[1]

[1] Department of Applied Health Research, University College London, London, UK

[2] Camden Borough Council, London, UK

[3] London Sport, London, UK

METHODS

Classification into low, moderate or high activity level

People were classified as moderately active if they had 3 or more days of vigorous-intensity activity of at least 20 minutes per day or 5 or more days of moderate-intensity activity and/or walking of at least 30 minutes per day or 5 or more days of any combination of walking, moderate-intensitiy or vigorous intensity activities achieving at least 600 MET (metabolic equivalent of task)-minutes/week. People were classified as highly active if they had vigorous-intensity activity on at least 3 days achieving at least 1500 MET-minutes/week or 7 or more days of any combination of walking, moderate-intensity or vigorous-intensity activities achieving at least 3000 MET-minutes per week. People not classified in the high or moderate activity level category were classified in the low activity level.

Baseline activity level of participants who completed the programme

In the main paper we present the baseline activity level of all recruited patients as well as the follow-up activity level of the completers. Table S1 shows the activity level at baseline for the completers only.

Table S1. Baseline activity level of participants who completed the programme

	Value	Source
**Baseline activity level - completers only**		
Low	27.2%	GIAG
Moderate	40.0%	GIAG
High	32.8%	GIAG
**Baseline activity level - completers with follow-up data**		
Low	31.4%	GIAG
Moderate	29.4%	GIAG
High	39.2%	GIAG
**Baseline activity level - completers without follow-up data**		
Low	24.3%	GIAG
Moderate	47.3%	GIAG
High	28.4%	GIAG

RESULTS

Disease incidence

Figure S1 shows the percentage of people in each health state over the first 25 years of the model. These percentages are almost identical for the Give it a Go programme and No intervention. After 25 years, 18.5% of people have died, almost 8% have coronary heart disease, almost 4% have had a stroke and 1.2% has diabetes, in both the ‘no intervention’ and the ‘Give it a Go’ population.

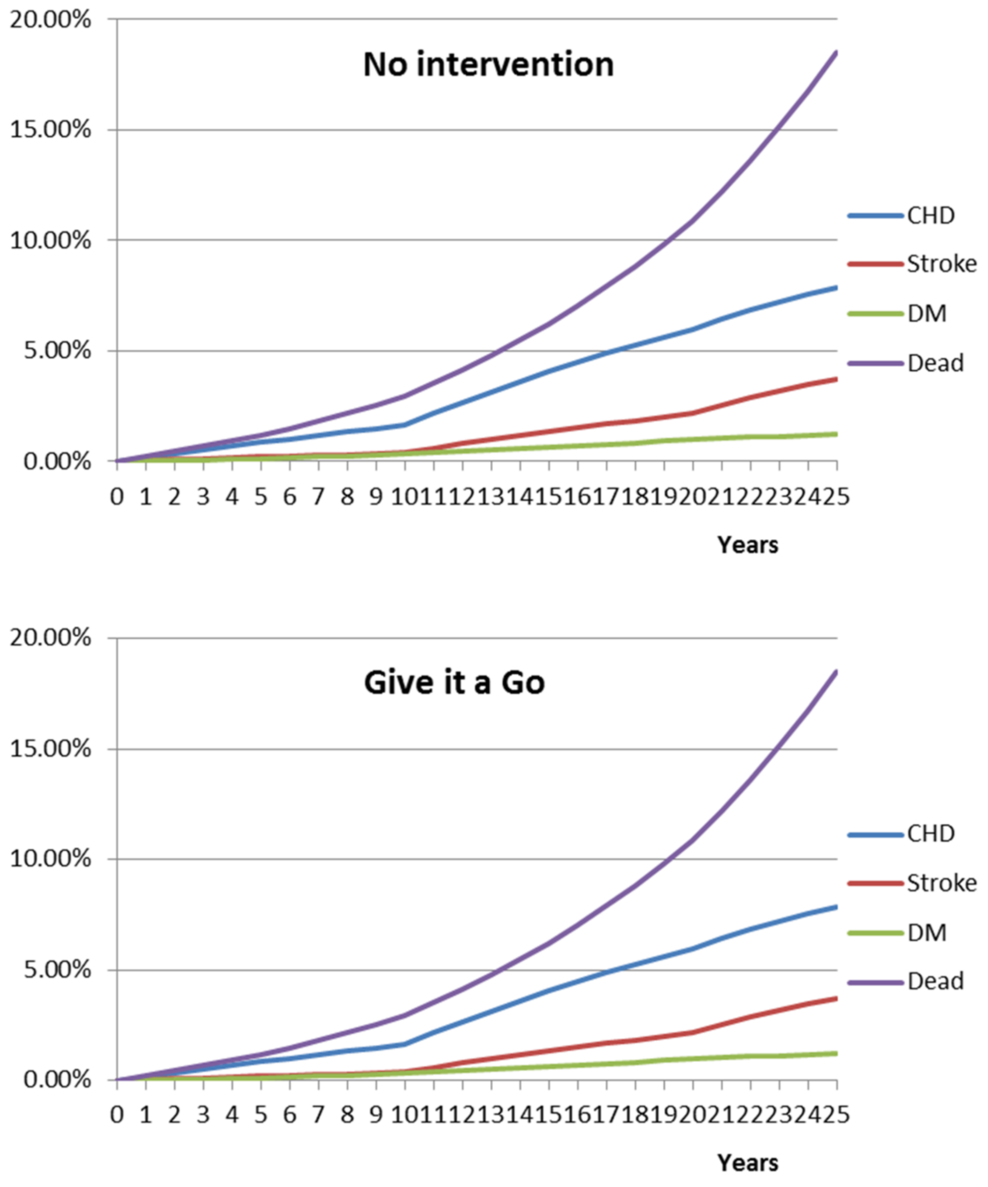

Figure S1. Proportion of people in the different health states during the first 25 years of the model who were recruited in the Give it a Go programme (total for completers and non-completers)
